# miR-302 regulates pluripotency, teratoma formation and differentiation in stem cells via an AKT1/OCT4-dependent manner

**DOI:** 10.1038/cddis.2015.383

**Published:** 2016-01-28

**Authors:** H-L Li, J-F Wei, L-Y Fan, S-H Wang, L Zhu, T-P Li, G Lin, Y Sun, Z-J Sun, J Ding, X-L Liang, J Li, Q Han, R-C-H Zhao

**Affiliations:** 1 Department of Cell Biology, Institute of Basic Medical Sciences Chinese Academy of Medical Sciences, School of Basic Medicine Peking Union Medical College, Tissue Engineering Center of Chinese Academy of Medical Sciences, Beijing, China; 2 Department of Histology and Embryology, School of Basic Medical Sciences, Xuzhou Medical University, Xuzhou, China; 3 Department of Obstetrics and Gynecology, Peking Union Medical College Hospital, Beijing, China; 4 Institute of Reproductive and Stem Cell Engineering, Key Laboratory of Stem Cells and Reproductive Engineering, Ministry of Health, Central South University, Changsha, China; 5 Department of Nuclear Medicine, Peking Union Medical College Hospital, Beijing, China; 6 Peking Union Medical College Hospital, Beijing, China

## Abstract

Pluripotency makes human pluripotent stem cells (hPSCs) promising for regenerative medicine, but the teratoma formation has been considered to be a major obstacle for their clinical applications. Here, we determined that the downregulation of miR-302 suppresses the teratoma formation, hampers the self-renewal and pluripotency, and promotes hPSC differentiation. The underlying mechanism is that the high endogenous expression of miR-302 suppresses the AKT1 expression by directly targeting its 3'UTR and subsequently maintains the pluripotent factor OCT4 at high level. Our findings reveal that miR-302 regulates OCT4 by suppressing AKT1, which provides hPSCs two characteristics related to their potential for clinical applications: the benefit of pluripotency and the hindrance of teratoma formation. More importantly, we demonstrate that miR-302 upregulation cannot lead OCT4 negative human adult mesenchymal stem cells (hMSCs) to acquire the teratoma formation *in vivo*. Whether miR-302 upregulation can drive hMSCs to acquire a higher differentiation potential is worthy of deep investigation.

Human pluripotent stem cells (hPSCs), including human embryonic stem cells (hESCs) and human-induced pluripotent stem cells, have the ability to self-renew and differentiate into cells of the three germ layers,^1, 2^ which makes such cells promising for regenerative medicine. However, upon transplantation *in vivo*, undifferentiated hESCs rapidly form stereotypical tumors called teratoma.^3^ The teratoma formation of hPSCs is a critical issue at present,^4, 5, 6^ and its cellular and molecular mechanisms remain largely unknown.

The remarkable self-renewal capacity of hESCs has been thought to be the parallel certain hallmarks of tumorigenesis,^5, 7^ which requires a unique cell cycle structure that is characterized by a shortened G1 phase and the absence of G1/S checkpoint.^8, 9^ The absence of cell cycle inhibitors^10, 11^ allows hESCs to proliferate rapidly and self-renew constantly.^8, 9^ ESCs share some cellular and molecular phenotypes with cancer cells.^6, 7, 12^ OCT4 is a core transcriptional factor for self-renewal and pluripotency in PSCs. OCT4 also express in some tumors,^13, 14, 15, 16^ and possess oncogenic potential^4, 17^ as a cell cycle promoter.^18^ These findings suggest that OCT4 may have important roles in the regulation of the cell cycle and tumorigenesis.

Today, it is widely recognized that in addition to protein-coding genes, microRNAs (miRNAs) are connected to the transcriptional regulatory circuitry of ESCs.^19, 20^ miR-302 can target both positive and negative cell cycle-related genes, increase the number of cells in S phase and shape an ESC-like cell cycle.^21^ ESCs cell-specific miRNAs including miR-302 can promote the G1 to S transition and rescue the proliferation defect in the Dgcr8 knockout ESCs.^22^ However, upregulation of miR-302 also suppressed CDK2 and CCND1, which simultaneously blocked the G1 to S transition in some tumor cells.^23^ OCT4 and SOX2 can bind to a conserved promoter region of miR-302^21^ and affect its expression in hESCs,^19, 21^ whereas miR-302 can induce and enhance somatic cell reprogramming to a pluripotent state.^24, 25, 26^ However, the role miR-302 has in the regulation of cell cycle, pluripotency and tumorigenicity in hPSCs and hMSCs remains unclear.

In this study, we comprehensively investigated the effects of miR-302 on the biological characteristics of hPSCs and hMSCs. We demonstrate that endogenous miR-302 in hPSCs is just a contributing factor for the pluripotency and the teratoma formation through promoting G1 to S transition and maintaining OCT4 at high level by directly inhibiting AKT1. However, although the expression of AKT1 was also sharply reduced and cell proliferation was accelerated by miR-302 upregulation in hMSCs, overexpression of miR-302 did not lead OCT4 negative hMSCs to acquire the ability of tumor formation, which suggested that the regulation of miR-302 on pluripotency and teratoma formation may be dependent on the high endogenous expression of OCT4 in cells.

## Results

### Downregulation of miR-302 inhibits the teratoma formation of hPSCs

**Figure 1:**
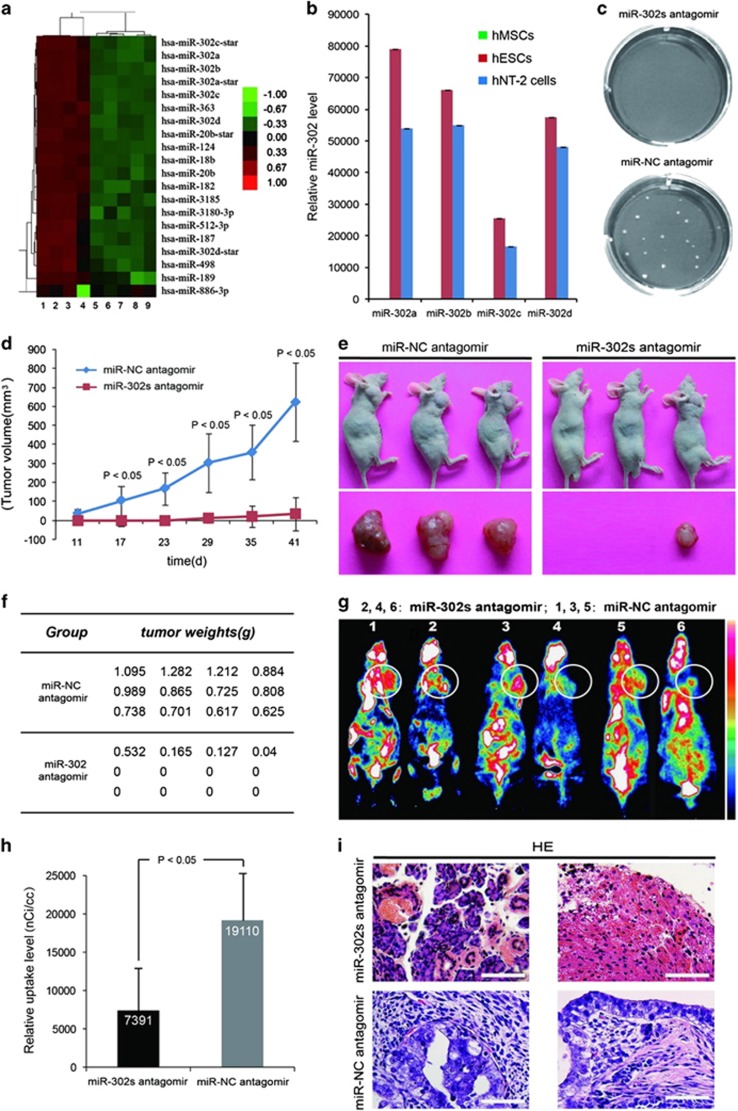
miR-302 downregulation inhibits the tumorigenicity of hPSCs *in vitro* and *in vivo*. (**a**) miRNA microarray analysis of the miRNA expression profile of hESCs, hMSCs, fibroblasts and embryoid bodies. 1–2: H9, 3: chHES-22, 4–6: hMSCs, 7: BJ fibroblast, 8: D2, 9: 15-day embryoid body. Expression values are represented in shades of red and green relative to being above (red) or below (green) the median expression value across all samples (log scale 2, from −3 to +3). (**b**) miR-302a, miR-302b, miR-302c and miR-302d expression are detected by miRNA-specific qRT-PCR assays (*n*=3). U6 was used as an internal normalization control, and data were normalized to the level of hMSCs. (**c**) Soft agar colony formation assay was performed using hNT-2 cells after transfection with miR-302s antagomir for 21 days. miR-NC antagomir-transfected hNT-2 cells was used as control. (**d**) miR-302s-suppressed hNT-2 cells were delivered into 6-week-old male athymic mice (BALB/c nu/nu strain) (*n*=12). The volume of tumors was calculated and plotted and tumor growth curves were obtained. Negative control-transfected hNT-2 cells were used as control. (**e**) Representative image shows tumor formation when hNT-2 cells were delivered into mice (BALB/c nu/nu strain). (**f**) The weights of the tumors were measured at the end of time. (**g**) Xenografts were scanned by small-animal PET. The numbers 2, 4, 6 indicate the miR-302s-suppressed hNT-2 cells group, and 1, 3, 5 indicate the negative control-transfected hNT-2 cells group. White circles showed the location of xenografts in the mice. Fifty minutes after FDG injection, mice were anesthetized by inhalation of 2% isoflurane in oxygen using Summit AS-1-000-7 animal isoflurane anesthesia equipment before microPET static data acquisition with continuous inhalation of isoflurane through an animal mask, and kept at 37 °C until imaging. White and black are relative to being above (white) or below (black) the uptake median expression value across all samples. (**h**) Tissue uptake value was expressed as radioactive count per milliliter (nCi/cc). Interscapular BAT maximum uptake values and xenograft mean uptake values are measured from sagittal images and coronal images respectively (*n*=6). The data are presented as the mean±S.D. (**i**) Histological analysis of xenografts was shown by hematoxylin and eosin staining. Scale bars, 100 *μ*m. *P-*values were obtained using Student's *t*-test

ESCs and human embryonal carcinoma cells (ECCs, a malignant counterpart of hESCs)^4^ have overlapping metabolic signatures,^27^ and similar gene, protein and miRNA expression profiles,^28, 29^ ECCs can serve as a model for studying the characteristics of ESCs.^30^ Human Tera-2 (hNT-2) cell is an ECC cell line that can differentiate into a variety of somatic tissues *in vivo*.^31^ We and other researchers confirmed that, hESCs and hNT-2 cells have the ability to form teratoma *in vivo* while hMSCs do not (Supplementary Figures S1A and D). Our miRNA microarray and TaqMan qRT-PCR data revealed that the endogenous expression levels of miR-302 family were high in hESCs and hNT-2 cells but very low in hMSCs (Figures 1a and b; Supplementary Figures S1E and F and Supplementary Table S1). We presumed that high endogenous expression of miR-302 in hPSCs might be responsible for their teratoma formation. miR-302s antagomir (miR-302a, miR-302b, miR-302c and miR-302d in combination) was used to silence the endogenous miR-302s *in vitro* and *in vivo* (Supplementary Figure S2A). We found that downregulation of miR-302s dramatically abrogated the colony formation ability of hNT-2 cells in soft agar (Figure 1c). The growth of tumors in miR-302s-downregulated xenografts was gradually delayed at different time points for up to 41 days after inoculation (Figure 1d). All mice produced teratocarcinomas in the negative control group, but only 25% of mice developed teratocarcinomas from miR-302s-downregulated cells and the tumor weights were decreased by 92% at the final time point (Figures 1e and f; Supplementary Figure S2B). Small-animal PET scans showed that xenografts of miR-302s-suppressed hNT-2 cells displayed smaller volumes and lower uptake of fluorodeoxyglucose (FDG) than those of negative control-transfected cells (Figures 1g and h). Differential maturation of liver and pancreatic tissue is visible in miR-302s-suppressed xenografts, which is a characteristic of well-differentiated and benign mature teratoma; while negative control cells formed mixed, poorly differentiated and malignant germ cell tumors (Figure 1i). Thus, miR-302 is able to promote the teratoma formation of hPSCs *in vitro* and *in vivo*.

Then miR-302-overexpressing lentiviral vector (lenti-miR-302s) that expresses a series of connected miR-302a/b/c/d sequences was used to upregulate the expression of miR-302s in hMSCs (Supplementary Figures S3A and B). *In vitro* anchorage-independent colony formation assay showed that no colony was formed either in miR-302s-overexpressed hMSCs or negative control cells. When miR-302s-overexpressed hMSCs were delivered into 6-week-old male athymic mice (BALB/c nu/nu strain) and immunodeficient nonobese diabetic/severe combined immunodeficiency (NOD/SCID) mice, all mice did not produce teratoma (data not shown). These results suggested overexpression of the miR-302s alone is not sufficient to lead hMSCs to acquire the ability of teratoma formation.

### miR-302 promotes the proliferation of pluripotent and adult stem cells

**Figure 2:**
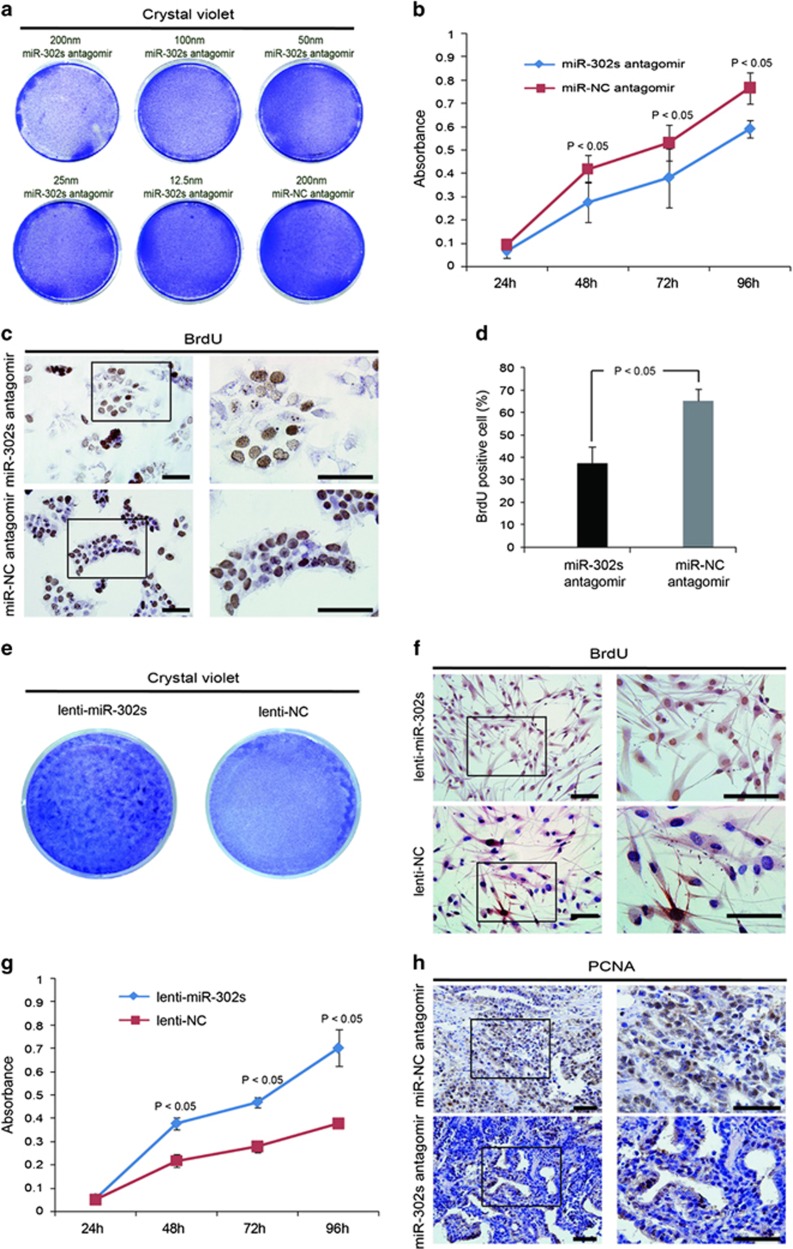
miR-302 promotes the proliferation of pluripotent and adult stem cells. (**a**) Crystal violet staining assessed the cell growth in different miR-302s antagomir concentration-treated hNT-2 cells. miR-NC antagomir was used as negative control. (**b**) The growth curve at different time points was obtained by the cell counting Kit-8. Data are presented as mean±S.D. (*n*=8). (**c**) Cell proliferation was compared by BrdU incorporation rates as revealed by immunocytochemical staining in miR-302s-downregulated hNT-2. Scale bars, 100 *μ*m. (**d**) Quantitative analysis of immunocytochemical staining for BrdU positive cells. BrdU positive cells were counted under a high power field and normalized by the total cell number. (**e**) The cell growth in miR-302s-upregulated hMSCs was assessed by crystal violet staining. miR-302s: miR-302-upregulated hMSCs by a miR-302-overexpressing lentiviral vector (miR-302a, miR-302b, miR-302c and miR-302d in combination), EGFP: a scrambled sequence infected hMSCs. (**f**) Immunocytochemical staining was used to detect the incorporation rates of BrdU in miR-302s-overexpressed hMSCs. Scale bars, 100 *μ*m. (**g**) The growth curve of miR-302s-overexpressed hMSCs at different time points was obtained by the cell counting Kit-8 assay. Lentivector expressing a scrambled sequence was used as a control. (**h**) The expression level of the proliferative index PCNA was investigated in the xenografts using an immunohistochemical staining method. Scale bars, 100 *μ*m. *P*-values were obtained using Student's *t*-test

Tumor formation is closely related to cell proliferation. Thus, we next analyzed the impact of miR-302 on the proliferation of hPSCs and found that cell growth was suppressed gradually with an increase in the concentration of miR-302s antagomir (Figure 2a). Downregulation of miR-302s resulted in the growth suppression and the BrdU incorporation rate decrease in hNT-2 cells at different time points (Figures 2b and d). Alkaline phosphatase (AP) staining assay showed that the inhibition of endogenous miR-302s resulted in the generation of smaller colonies from hESCs (Supplementary Figures S3C and D). In addition, upregulation of miR-302s in hMSCs accelerated cell growth and proliferation (Figures 2e and g; Supplementary Figure S3E). The expression level of proliferative marker PCNA was significantly reduced in the xenografts generated from miR-302s-suppressed hNT-2 cells (Figure 2h). These results demonstrated that miR-302 can promote cell proliferation both in pluripotent and adult stem cells.

### miR-302 dominantly regulates a set of cell cycle inhibitors and promotes the G1 to S transition

**Figure 3:**
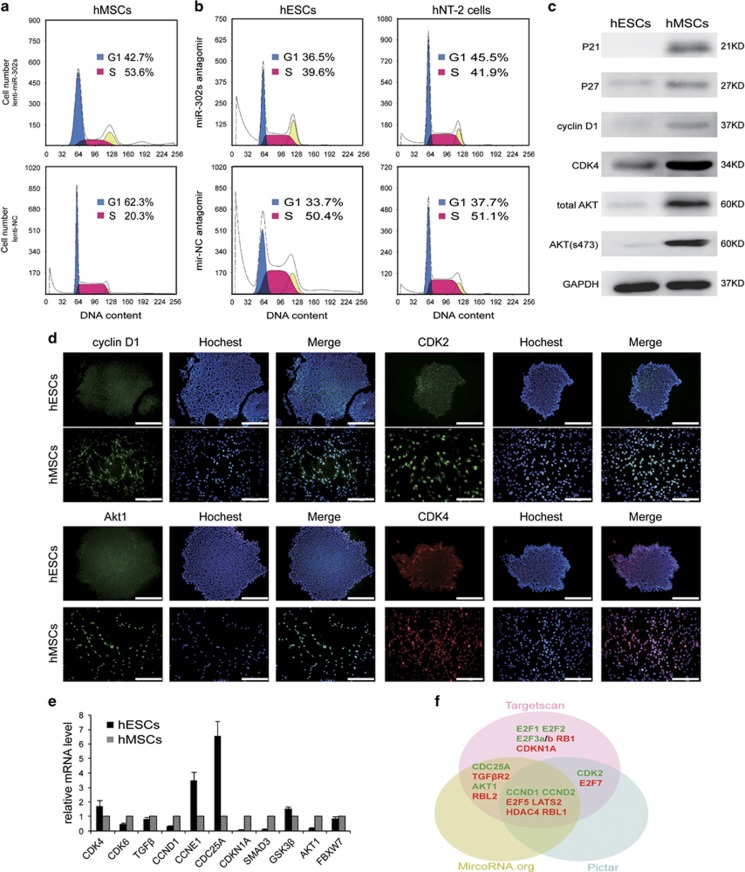
miR-302 regulates cell cycle progression by promoting the G1 to S transition. (**a**) Flow cytometry analysis of the cell cycle distribution in hMSCs when exogenous expression of miR-302 was upregulated. A lentiviral vector expressing a scrambled sequence was used as a control. (**b**) Flow cytometry analysis of the cell cycle distribution in hESCs and hNT-2 cells when endogenous expression of miR-302s was suppressed by miR-302s antagomir. Negative control oligonucleotide antagomir was transfected as control. (**c**) Western blot analysis in hESCs and hMSCs of the expression levels of key regulators involved in the G1 to S transition; GAPDH was used as a loading control. (**d**) Immunofluorescence detected the expression of proteins by Hoechst 33342 counterstaining. Scale bars, 200 *μ*m. (**e**) qRT-PCR measured the mRNA expression of key regulators involved in the G1 to S transition. GAPDH was used as an internal normalization control. Data are presented as mean±S.D. (*n*=3) and are representative of three independent experiments. (**f**) Targetscan, PicTar and Miranda predicated the cell cycle-associated candidate targets of miR-302. Green indicated positive regulators, red indicated negative regulators

Short G1 phase and fast G1 to S transition lead to the proliferation and teratoma formation of ESCs and iPSCs.^5, 32^ To explore the intrinsic mechanisms underlying the regulation of teratoma formation by miR-302, we analyzed the cell cycle distribution and the expression patterns of key cell cycle regulators associated with the G1 to S transition in hESCs, hNT-2 cells and hMSCs. Flow cytometry revealed that 50% of hESCs and hNT-2 cells were in S phase, while only 30% of hMSCs were in S phase (Supplementary Figure S4A). Then, we suppressed the endogenous expression of miR-302s in hESCs and hNT-2 cells, and increased its expression in hMSCs. Results showed that the percentage of cells in S phase increased from 20.3 to 53.6% (Figure 3a) in miR-302s-overexpressed hMSCs, while it decreased from 50.4% and 51.1% to 39.6% and 41.9% in miR-302s suppressed-hESCs and -hNT-2 cells, respectively (Figure 3b). These results indicated that miR-302 promotes cell growth and proliferation partially through promoting the G1 to S transition.

We then analyzed the expression levels of key regulators in the G1 to S transition and found that CDK2, CDK4, total AKT and phosphorylated AKT at S473 and P27 were at lower levels, and Cyclin D1 and P21 were almost absent in hESCs, compared with hMSCs (Figures 3c and e). We speculated that miR-302 might be involved in maintaining the low expression levels of these genes in hESCs. TargetScan, PicTar and Miranda suggested that many genes including both positive and negative G1 to S transition-associated regulators are candidate targets of miR-302 (Figure 3f). Previous evidence has demonstrated that the cell cycle promoters CCND1 and CDK2, and the inhibitors CDKN1A, RB1, RBL1, RBL2, LATS2 and TGFBR2, are direct targets of miR-302.^21, 22, 23, 33, 34, 35^ Consistent with these results, we revealed that the expression levels of CCND1, CDK4, CDK6, CDKN1A and TGF*β* were increased in miR-302 downregulated-hNT-2 cells and decreased in miR-302 upregulated-hMSCs (Supplementary Figures S4B and C). Our findings suggested that miR-302 can enhance proliferation through the dominant regulation of a set of cell cycle inhibitors, and result in rapid G1 to S transition.

### miR-302 regulates pluripotency by promoting self-renewal and suppressing differentiation

**Figure 4:**
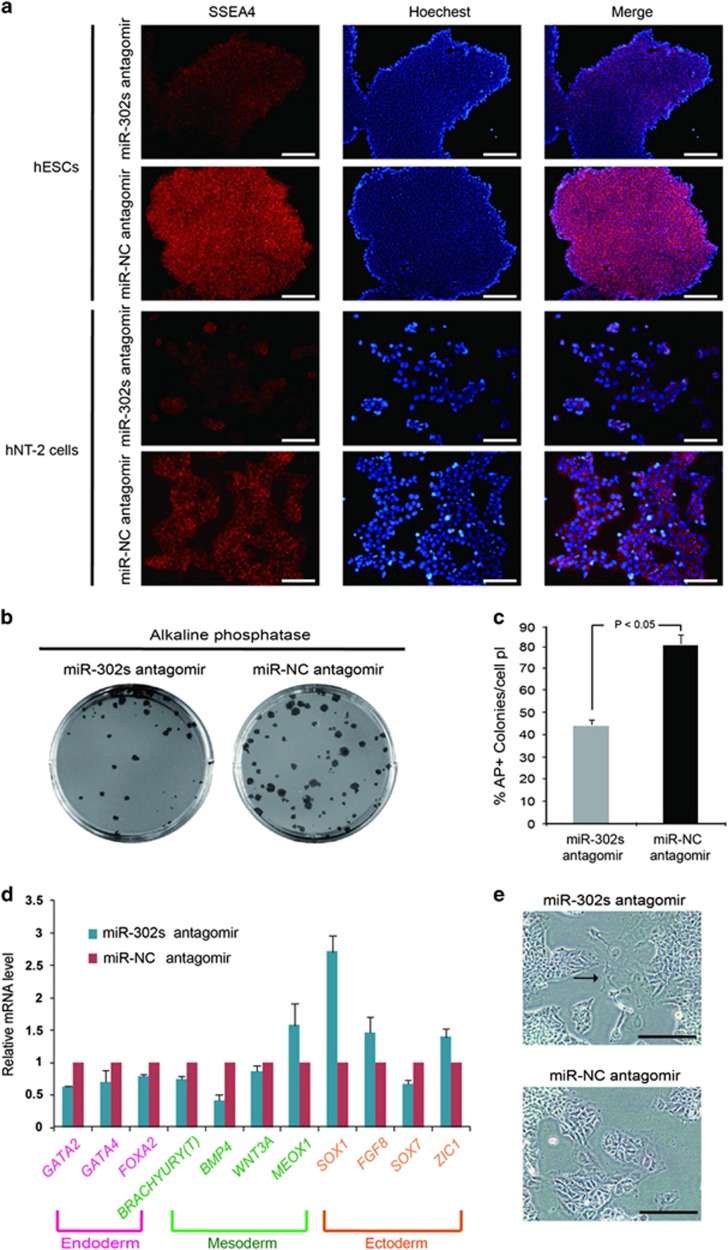
Downregulation of miR-302 suppresses self-renewal and promotes differentiation. (**a**) Immunofluorescence staining of SSEA4 in hESCs and hNT-2 cells when endogenous miR-302 was inhibited by miR-302s antagomirs. The negative control oligonucleotide antagomir was transfected as a control. Scale bars, 200 *μ*m. (**b**) miR-302s-downregulated hESCs were cultured in non-adherent conditions to form EBs. EBs were then dissociated and plated back in ESC culture conditions at day 15. ESC-like colonies were analyzed by AP staining after 6 days under EB to ESC conditions. (**c**) The numbers of AP-positive ESC-like colonies were calculated under EB to ESC conditions in miR-302s-downregulated hESCs. Negative control oligonucleotide antagomir was transfected as control. Data are presented as mean±S.D. (*n*=5) and are representative of three independent experiments. (**d**) hNT-2 cells were treated with vehicle or 1 mM retinoic acid (RA) for 4 days and the expression levels of lineage-specific markers were determined by qRT-PCR; GAPDH was used as an internal normalization control. Data are presented as mean±S.D. (*n*=3) and are representative of three independent experiments. (**e**) Morphological changes of hNT-2 cells were investigated after treatment with vehicle or 1 mM retinoic acid (RA) for 4 days in miR-302s antagomir-transfected hNT-2 cells and negative control cells. Scale bars, 100 *μ*m. *P*-values were obtained using Student's *t*-test

Taking into account the positive feedback regulation involved in the G1 to S transition, self-renewal and pluripotency in hPSCs,^5, 36, 37^ we further assessed the role of miR-302 in self-renewal and pluripotency. According to the Protocol Exchange (Patel S, Pine S, Rameshwar P. Noble Agar Assay for Self-Renewal. Protocol Exchange 2013), single-cell clonogenic assay also indicated the self-renewal ability of stem cells. As mentioned above, our single-cell clonogenic assay revealed that downregulation of endogenous miR-302s significantly inhibited the self-renewal (Figure 1c). Immunofluorescence analysis further indicated that the expression of self-renewal marker SSEA4 was decreased in miR-302s—downregulated hPSCs (Figure 4a).

Self-renewal involves proliferation with a concomitant suppression of differentiation.^8^ Therefore, to further analyze the effects of miR-302 on the modulation of differentiation, hESCs were subjected to EB formation and then placed back into ESC conditions. shRNA was used to suppress endogenous expression of miR-302s and to investigate the function of miR-302 on pluripotency and differentiation of hPSCs. Results showed that downregulation of endogenous miR-302s resulted in fewer AP-positive colonies in hESCs (Figures 4b and c). We next investigated whether miR-302 has a similar role in hNT-2 cells. Retinoic acid (RA) was employed to induce the differentiation.^21^ Results showed that most endoderm- and mesoderm-associated markers were suppressed while ectodermal and neural differentiation-related genes were upregulated in miR-302s-downregulated hNT-2 cells (Figure 4d), which accompanied with a flattened cell morphology indicating the differentiation of cells (Figure 4e). These results showed that the inhibition of miR-302 negatively impacted self-renewal and pluripotency and promoted the differentiation of hPSCs.

### miR-302 can directly target and regulate the expression of AKT1 in pluripotent and adult stem cells

**Figure 5:**
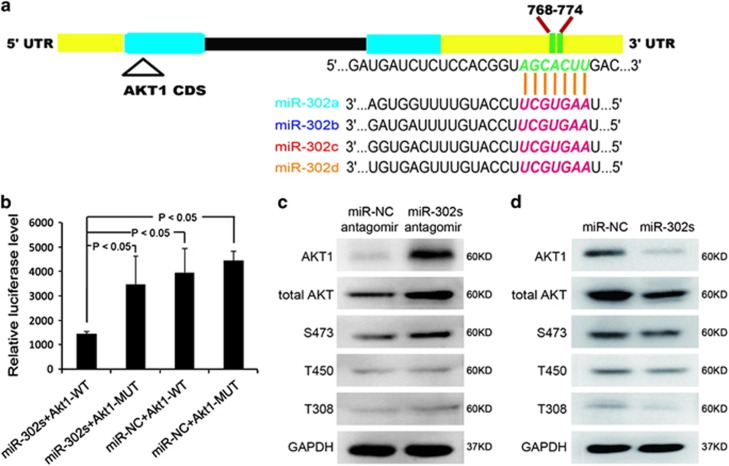
miR-302 represses the expression of AKT1 by targeting its 3'UTR. (**a**) Bioinformatics analysis showed that there is a 7-bp sequence in the 3'UTR of AKT1 that is complementary to the seed sequence of miR-302. (**b**) Luciferase activity was assessed in miR-302s mimic-transfected 293 T cells. Negative control oligonucleotide was used as control. Data are presented as mean±S.D. (*n*=3) and are representative of three independent experiments. WT, wild type; MUT, mutant-type. (**c**, **d**) Western blot detected the expression levels of total AKT, AKT1 and phospho-AKT in miR-302s-antagomir-transfected hNT-2 cells and miR-302s-upregulated hMSCs. A negative control oligonucleotide was transfected as control, and GAPDH was used as a loading control

AKT1 has been implicated to be an important regulator of self-renewal and pluripotency.^38, 39^ We found that miR-302 could regulate the expression of AKT1 (Supplementary Figures S4B and C). Thus, we speculated that AKT1 might be one of the direct targets of miR-302 that controls the self-renewal and pluripotency of hPSCs. Bioinformatics analysis showed that there is a 7-bp sequence within the 3'UTR of AKT1 that is complementary to the sequence of miR-302 (Figure 5a). To further validate whether miR-302 can directly target AKT1, we designed dual luciferase reporter gene vectors containing either wild type (AKT1-WT) or mutated (AKT1-MUT) putative 3′UTR sequences for miR-302-binding (family ‘seed sequence') and inserted them into the 3′UTR of the luciferase genes. Results displayed that the overexpression of miR-302 significantly suppressed >58% of the reporter luciferase activity of the AKT1-WT reporter plasmid but not that of the AKT1-MUT reporter plasmid, suggesting a direct targeting relationship between miR-302 and the 3'UTR of AKT1 (Figure 5b). qRT-PCR and western blot analyses showed that miR-302 can regulate the expression of total AKT1 and phosphorylated AKT1 mainly at S473 locus (Figures 5c and d; Supplementary Figures S4B and C) both in hNT-2 and hMSCs. These results strongly confirmed that miR-302 can directly target AKT1 and suppress its expression.

### miR-302 contributes to the pluripotency and teratoma formation of hPSCs by maintaining OCT4 expression via suppressing AKT1

**Figure 6:**
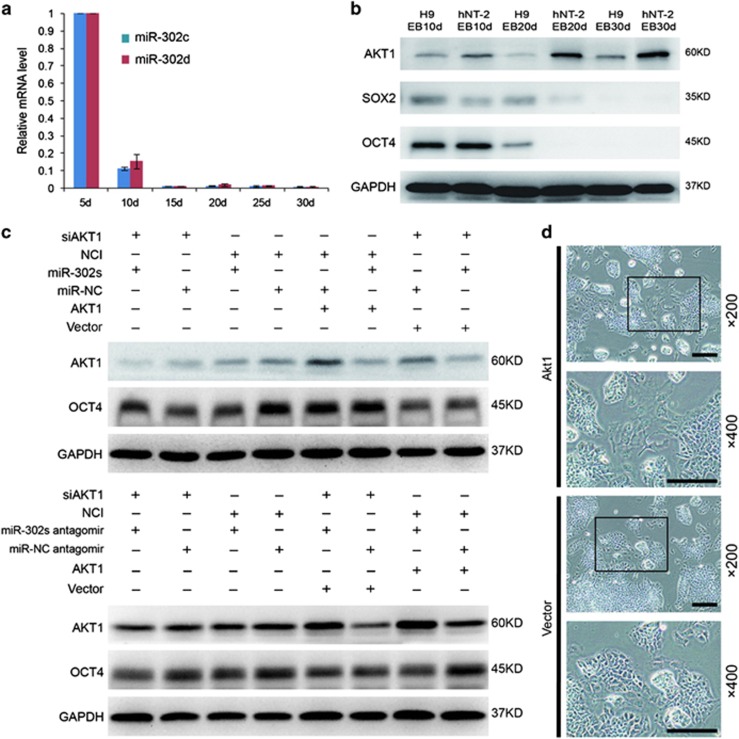
miR-302 contributes to the pluripotency and tumorigenicity in hPSCs by maintaining OCT4 at high expression level through suppressing of AKT1. (**a**) The expression of miR-302s was analyzed during the process of EB formation at days 5, 10, 15, 20, 25 and 30 by TaqMan qRT-PCR assays. U6 was used as an internal normalization control. Data are presented as mean±S.D. (*n*=3) and are representative of three independent experiments. (**b**) The expression of AKT1, OCT4 and SOX2 were assessed by western blot during the differentiation of hESCs and hNT-2 cells to EBs. GAPDH was used as a loading control. (**c**) The western blot analysis evaluated the expression of AKT1 and OCT4 when miR-302 was upregulated by transfected miR-302s mimics in AKT1 overexpressing hNT-2 cells. A negative control oligonucleotide was transfected as control, and GAPDH was used as a loading control. (**d**) Western blot analysis was used to evaluate the expression of AKT1 and OCT4. A negative control oligonucleotide antagomir was transfected as a control, and GAPDH was used as a loading control. (**e**) AKT1 overexpressing vectors were transfected into hNT-2 cells for 5 days, and the morphological changes of AKT1-overexpressed hNT-2 cells and negative control cells were observed under light microscope. Scale bars, 100 *μ*m

**Figure 7:**
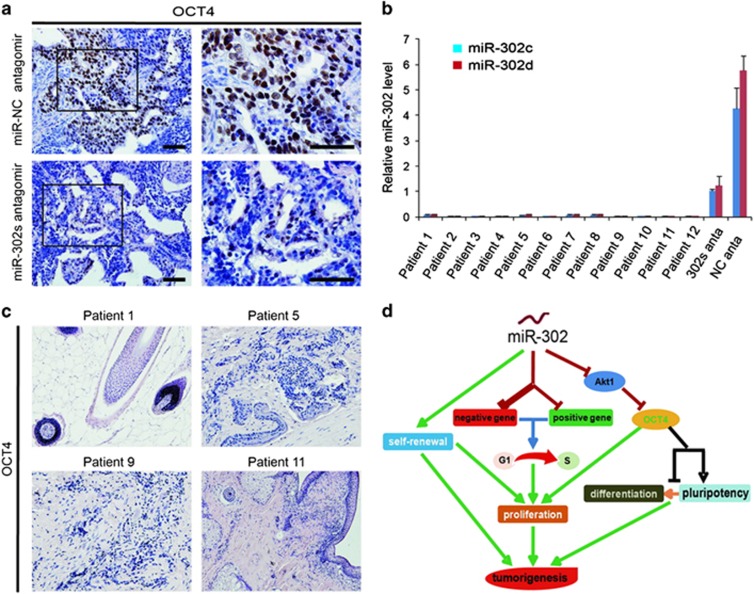
The expression levels of miR-302 and OCT4 in highly differentiated patient-derived teratoma and hNT-2 cells generated malignant teratoma xenografts. (**a**) The immunohistochemical analysis of OCT4 in miR-302s-suppressed hNT-2 cells xenografts. Negative control oligonucleotide-transfected hNT-2 cells xenografts as control. Scale bars, 100 *μ*m. (**b**) The expression of miR-302 in 12 highly differentiated teratoma patients' specimens by TaqMan qRT-PCR analysis. RNU48 was used as an internal normalization control. The data are presented as the mean±S.D. (*n*=3) and are representative of three independent experiments. miR-302s antagomir: miR-302s-suppressed hNT-2 cell xenografts, miR-NC antagomir: miR-NC-transfected hNT-2 cell xenografts. (**c**) The expression of OCT4 was detected in 12 highly differentiated teratoma specimens by immunohistochemistry. Scale bars, 100 *μ*m. (**d**) The pathway by which miR-302 contributes to the tumorigenicity and the pluripotency of hPSCs through the dominant regulation of the G1 to S transition and the maintenance of the pluripotency factor OCT4 at high level via suppressing AKT1

To clarify whether miR-302 mediates pluripotency and differentiation of hPSCs through the regulation of AKT1, we first analyzed the expression of miR-302 during embryoid body (EB) formation.^20^ We found that miR-302 was gradually downregulated during EB differentiation (Figure 6a), while at the same time, AKT1 was gradually upregulated, OCT4 and SOX2 were downregulated (Figure 6b). The positive correlation between miR-302 and OCT4, and negative correlation between miR-302 and AKT1 suggested that miR-302 may enhance the expression of pluripotency regulators through suppressing AKT1. To clarify this hypothesis, the AKT1-overexpressing vectors were transfected into the hNT-2 cells. Results showed that OCT4 was decreased in AKT1-overexpressed hNT-2 cells, while its expression was increased in AKT1 suppressed-hNT-2 cells and was accompanied with morphological changes similar to the effect of miR-302 on the expression of AKT1 and OCT4 (Figures 6c and e). In addition, the upregulation of miR-302 in AKT1-overexpressed hNT-2 cells could inhibit AKT1 and rescue the expression of OCT4. Furthermore, miR-302 upregulation could not significantly change the expression level of OCT4 in siAKT1-transfected hNT-2 cells (Figures 6c and d), while repression of miR-302 led to the opposite effects. These results strongly demonstrated that miR-302 can maintain the expression of OCT4 at high level, at least in part by repressing AKT1. In addition, the expression of OCT4 was markedly reduced in teratoma generated from miR-302-downregulated hNT-2 cells (Figure 7a). Moreover, the expression of miR-302 is very low or almost undetectable in highly differentiated patient-derived teratoma as compared with hNT-2 cells generated malignant teratoma xenografts (Figure 7b). Similarly, the expression of OCT4 was also undetectable in these 12 highly differentiated teratoma patient's specimens (Figure 7c). Therefore, our findings revealed that high expression levels of endogenous miR-302 in hPSCs are beneficial for the pluripotency and tumorigenicity of hPSCs by maintaining OCT4 at high level through suppressing AKT1 (Figure 7d). Overexpression of miR-302 also significantly decreased AKT1 expression in hMSCs (Supplementary Figure S5A), whereas OCT4 always maintained at undetectable level regardless of the expression changes of miR-302 and AKT1 in hMSCs (Supplementary Figures S6A and B).

## Discussion

miRNAs are important epigenetic regulatory molecules, have been demonstrated to participate in many biological processes.^40^ Here, we demonstrate that miR-302 has an important role in regulating cell proliferation, self-renewal, pluripotency, differentiation and teratoma formation. miR-302 can promote the proliferation and self-renewal both in hPSCs and hMSCs through dominantly regulating a set of cell cycle inhibitors and accelerating the G1 to S transition. High expression of endogenous miR-302 in hPSCs is a contributing factor for the pluripotency and teratoma formation through maintaining OCT4 at high level by directly inhibiting AKT1. However, upregulation of miR-302 cannot lead OCT4 negative hMSCs to acquire the teratoma formation.

hPSCs have strong teratoma formation ability and high endogenous expression of miR-302.^24, 41, 42, 43, 44^ However, there is a big controversy about what role miR-302 has in the regulation of stemness, cell proliferation, tumorigenesis and differentiation. In some cases, miR-302s have been shown to produce iPSCs from human somatic cells and cancer cells.^25, 26, 45, 46, 47^ Alternatively, miR-302s also have been reported to inhibit the tumorigenicity of hPSCs and various cancer cells.^23, 48, 49^ We revealed that downregulation of miR-302 significantly decreased the rate of tumor formation and reduced the tumor volume in hPSCs.

Previous studies have indicated that tumorigenicity is closely related to self-renewal and cell cycle.^5, 7, 8, 9, 33, 35^ To date, both positive and negative cell cycle-associated factors have been reported as direct targets of miR-302.^21, 22, 23, 33, 34, 35^ We and several reports demonstrated that miR-302s downregulated CDK2, CDK4, AKT1, P27, cyclin A, E2F-1 and cyclin D1^21, 23, 50^ in stem cells, while others reported that the levels of CDK2, cyclin A and E2F-1 were unaffected in miR-302-367-overexpressed HeLa and SiHa cells.^51^ We found a marked suppression of cell cycle progression in miR-302-suppressed-hNT-2 cells, while an increase in the number of S phase cells in miR-302-overexpressed hMSCs. This result coincides with recent report that miR-302 induces proliferation in human adipose tissue-derived MSCs.^52^ Thus, our data suggest that the negative cell cycle regulators are dominant targets of miR-302 in ESCs, hNT-2 cells and hMSCs.

The ectopic expression of miR-302 is able to reprogram and promote human somatic cells to ESC-like cells.^53, 54^ Recently, the role of miR-302 in nerve development has been reported. NPCs exhibit enhanced proliferation, precocious differentiation and decreased cell death^55^ or survival^56^ in miR-302s mutant embryos. More recently, miR-302 has been reported to be capable of regulating Brg1 chromatin remodeling complex composition in hESCs, and subsequently regulating pluripotency by positively influencing mesendodermal differentiation.^57^ These findings suggested a complicated relationship between pluripotency and differentiation related to miR-302. We found that inhibition of miR-302 causes a significant decrease in self-renewal ability and promotes differentiation accompanied by downregulation of OCT4, suggesting that high level of endogenous miR-302 may be responsible for the maintenance of self-renewal and pluripotency in hPSCs.

Some reports implied that abnormal activation of AKT negatively regulates HSC stemness.^58, 59, 60^ The functions of AKT in stem cells^59, 61, 62^ have been investigated, but its precise role in the mechanism by which AKT modulates stemness and differentiation is not yet clarified. Here, we observed that in addition to directly targeting cell cycle-associated genes, miR-302 also affects the expression of total AKT1 and phospho-AKT mainly at S473 locus. We confirmed that AKT1 is a direct target of miR-302 in the regulation of pluripotency and differentiation of hPSCs.

Recently, AKT was found to phosphorylate OCT4, which facilitated OCT4's stability, nuclear localization and interaction with SOX2, subsequently promoted the transcription of OCT4 and NANOG. On the other hand, Akt phosphorylates OCT4 and KLF4, promoting their degradation via the ubiquitin–proteasome system.^63^ More recently, AKT1 was found to boost the function of differentiation factor SATB1 yet attenuate OCT4/KLF4 activity by phosphorylating SATB1 in ECCs.^63^ Consistent with this report, we found that downregulation of endogenous miR-302s or overexpression of AKT1 significantly decreased the Oct4's protein level, inhibited the self-renewal and promoted differentiation in hPSCs. Upregulation of miR-302 in AKT1-overexpressed hPSCs could inhibit AKT1 and rescue the protein level of OCT4. These results revealed that miR-302 indirectly regulates OCT4 protein level by suppressing AKT1 and subsequently avoiding OCT4's degradation.

In addition, OCT4 which is continuously required to maintain ESC pluripotency, has been identified to be expressed in some tumors^13, 14, 64, 65^ and is taking part in the regulation of the cell cycle by accelerating the G1 to S transition.^18^ We found that OCT4 was markedly reduced in teratoma generated from miR-302-downregulated hNT-2 cells, which is in agreement with our results *in vitro*. Importantly, in highly differentiated teratoma of patients, which are more common clinically, the expression of miR-302 is very low or even undetectable, and the expression of OCT4 is still undetectable.

In summary, our findings first uncover that miR-302 indirectly regulates OCT4 by suppressing AKT1, which provides hPSCs two characteristics related to their potential for clinical applications: the benefit of pluripotency and the hindrance of teratoma formation. Overexpression of miR-302 did not lead OCT4 negative hMSCs to express OCT4 and acquire the teratoma formation, which suggested that the regulation of miR-302 on pluripotency and teratoma formation by direct suppression of AKT1 may be dependent on the high endogenous expression of OCT4 in cells. Whether upregulation of miR-302 can drive hMSCs to acquire a higher differentiation potential is worthy of deep investigation.

## Materials and Methods

### Cell lines and cell culture

hESCs cell lines H9 (Wicell, Madison, WI, USA) and chHES-22 were grown on mitomycin C (Sigma Aldrich, St. Louis, MO, USA) inactivated mouse embryonic fibroblasts (MEFs) in medium containing Dulbecco's modified Eagle's medium F-12 (DMEM; Gibco, Raritan, NJ, USA) supplemented with 20% knockout serum replacement (Gibco), 1 mM l-glutamine (Gibco), 0.1 mM *β*-mercaptoethanol (Gibco), 1% nonessential amino acids (Gibco) and 4 ng/ml human basic fibroblast growth factor (bFGF) (Sigma Aldrich). One to two passages before beginning our experiments, hESCs were transferred to Matrigel (BD Biosciences)-coated plates with mTeSR (Stem Cell, Vancouver, British Columbia, Canada). Differentiation through EB formation was induced by collecting hESCs and culturing them in suspension with hESC medium without bFGF. The hNT-2 cell line was obtained from the cell culture center of the Institute of Basic Medical Sciences of the Chinese Academy of Medical Sciences and the School of Basic Medicine of the Peking Union Medical College. hNT-2 cells were cultured according to the literature.^23^ Bj (ATCC, Maryland Rockefeller, MD, USA, CRL-2522) and D2 fibroblasts were cultured in medium consisting of DMEM supplemented with 10% fetal bovine serum, and digested using trypsin-EDTA (Gibco). Cell cultures were maintained at 37 °C in a humidified incubator with 5% CO_2_. hNT-2 cells gave rise to embryonic bodies (EBs) in suspension culture. Differentiation by forming EB suspension was performed in hNT-2 cells as report.^20^

### Isolation and expansion of human MSCs

hMSCs were isolated from adipose tissues that were obtained from patients undergoing tumescent liposuction according to procedures approved by the Ethics Committee at the Chinese Academy of Medical Sciences and Peking Union Medical College. Human specimens were digested in collagenase P (Roche, Mannheim, Germany) for 30 min at 37 °C and centrifuged at 300 × g for 5 min. Then, the pellet was suspended in media consisting of DF12 (Gibco) supplemented with 5% MSC fetal bovine serum (Gibco). After 6±1 days in culture at 37 °C in a humidified incubator with 5% CO_2_, adherent cells were selected for phenotypic analysis of hMSCs using flow cytometry. The expression of CD73, CD90 and CD105, together with a lack of expression of CD11b, CD14, CD34, CD45, CD19 and HLA-DR cell population was defined as mesenchymal stem cells (Supplementary Figures S6).^66^

### Expression profiling with microarrays

Microarray experiments were conducted according to the manufacturer's instructions (www.capitalbio.com). Total RNA was prepared with TRIzol Reagent (Invitrogen, Paisley, UK). miRNA was extracted by using a mirVana miRNA Isolation Kit (Ambion, Austin, TX, USA). RNA integrity was determined by 1% formaldehyde denaturing gel electrophoresis. miRNA profiling was performed using GeneChip miRNA Array version 2.0 (Affymetrix, Santa Clara, CA, USA). Eukaryotic Hybridization Control Kit, Hybridization, Wash and Stain Kit are used to perform Hybridization. After labeling, the samples were hybridized on Affymetrix Gene-Chip miRNA arrays, washed, stained and scanned according to the manufacturer's instructions, the images obtained were then analyzed with LuxScan 3.0 software (both from CapitalBio, Beijing, China). Raw data were performed background adjustment, normalized using the rank invariant gene, and filtered for significant expression on the basis of negative control beads.

### Lentiviral preparation and infection

Lentivirus production and titering were carried out according to the protocols from invitrogen (http://www.invitrogen.com.cn). The cells were passaged in every 2 days using 0.05% trypsin-EDTA to single cell and plated in 2 ml aliquots per well in six-well plates at multiplicity of infection (MOI) 30. After the cells were infected with the miR-302 overexpressing GFP-labeled lentiviral vector (lenti-miR-302s), GFP-positive cells were sortinged by FACSCalibur (BD). The same GFP-labeled lentiviral vector expressing a scrambled sequence (lenti-NC) with no homology to the human genome was used in parallel as a control.

### RNA extraction and real-time PCR assays

Total RNA was extracted from cultured cells or freshly iced teratoma tissues with TRIzol Reagent (Invitrogen). The mRNA levels were assayed with the primer sets (see Supplementary Table S2 in the Supplementary Material) in an Applied Biosystems Fast Real-Time PCR System (Applied Biosystems, Foster, CA, USA) according to the manufacturer's instructions. miRNA was extracted with the miRVana Isolation Kit (Ambion) as described by the manufacturer. The miRNA levels were assayed by TaqMan Small RNA Assays using probe sets or SYBR green-based stem-loop technology (see Supplementary Table S3 in the Supplementary Material) designed to detect and quantify mature miRNAs (Invitrogen); the procedure was done according to the manufacturer's instructions.

### Western blot analysis

After washing twice with PBS, proteins were extracted with radioimmunoprecipitation lysis buffer with PMSF (Beyotime, Shanghai, China) and quantified by the BCA Protein Assay Kit (Beyotime). Lysates were electrophoresed on a 10% SDS-PAGE gel and electrophoretically transferred to a polyvinylidene difluoride membrane. The membranes were blocked in Tris-buffered saline with 5% milk and 0.1% Tween. The blots were probed with CDK4, Cyclin D1 (1 : 200; all from Santa Cruz Biotechnology, Inc., Delaware, Santa Cruz, CA, USA), P21, P27, total AKT, AKT1, Phospho-AKT (S473, T450, T308), OCT4, SOX2 (1 : 1000; all from Cell Signaling Technology, Danvers, MA, USA) and GAPDH (Sigma Aldrich) at 4 °C overnight and revealed with anti-mouse or anti-rabbit horseradish peroxidase-conjugated secondary antibodies (1 : 2000; ZSbio). Antibody–antigen complexes were detected using the chemiluminescent ECL reagent (Millipore, Billerica, MA, USA). Western blot images were acquired using an Alpha Innotech FluorChem SP Imaging System (Alpha Innotech, San Jose, CA, USA).

### Dual luciferase reporter assay

An 80–100 bp synthetic fragment of gene 3'UTR containing the predicted seed match site or the mutant site technology (Supplementary Table S4) was inserted between the NotI and XbaI cleavage sites of the pRL-TK report vector (Promega, Southampton, UK) downstream from the renilla luciferase reporter gene. In all, 5 × 10^4^ 293 T cells per well in 24-well plates were co-transfected with 1 *μ*g pRL-TK vector with or without the synthetic fragment of the AKT1 3′UTR and 0.1 *μ*g pGL-3 control vector with firefly luciferase reporter gene, and 100 nM miR-302 mimics or miR-NC mixed with Lipofectamine 2000 (Invitrogen), respectively, according to the manufacturer's instructions. Luciferase activity was measured in triplicate by using the Dual Luciferase Reporter Assay System (Promega). Renilla luciferase activity was normalized to firefly luciferase activity.

### Transfection

Cells were transfected by FuGENE HD (Roche) or Lipofectamine 2000 (Invitrogen) according to the manufacturer's instructions. Cells were transfected with 200 nM of miR-302s antagomir (50 nmol each of miR-302a antagomir, miR-302b antagomir, miR-302c antagomir and miR-302d antagomir), 200 nM miR-302s mimics (50 nmol each of miR-302a, miR-302b, miR-302c and miR-302d) or 200 nM negative control NC or 200 nM negative control antagomir as a control (Genepharma, Shanghai, China) (see Supplementary Table S5 in the Supplementary Material). Three pairs of siRNA for AKT1 and pIRES2-EGFP-AKT1 Vector (NM_005163.2) were designed and synthesized (Invitrogen). The most efficient siRNA for AKT1 were transfected into cells (see Supplementary Table S6 in the Supplementary Material) with the same procedure as miRNA transfection.

### Cell cycle analysis

Cell cycle analysis was performed using the CycleTest plus DNA reagent kit (BD). Cells were collected by typsin treatment and counted with a hemocytometer. A total of 500 000 cells per well were fixed, permeabilized and stained in accordance with the manufacturer's instructions, and the sample was analyzed by flow cytometry using a COULTER EPICS XL. Data were analyzed using MultiCycle software to generate the percentages of cells in G1, S and G2 to M phases of the cell cycle.

### BrdU incorporation assay

Cells were incubated in a medium containing 30 *μ*g/l BrdU (Sigma Aldrich) for 45 min at 37 °C in a humidified atmosphere (5% CO_2_). Cells were fixed with ethanol and 50 mM glycine, pH 2.0, for 45 min at room temperature and denaturated in 4 M HCl for 10 min. The subsequent detection of BrdU was accomplished with antibodies for BrdU (1 : 5000) (Sigma Aldrich). The next steps were performed according to the manufacturer's instructions using an ABC detection kit (Vector Laboratories, Burlingame, CA, USA).

### Alkaline phosphatase staining

AP staining was performed using an Alkaline Phosphatase Detection kit (Millipore) according to the manufacturer's instructions. Cells were fixed in 90% methanol and 10% formamide for 2 min and washed once with rinse buffer (20 mM Tris-HCl, pH 7.4 and 0.05% Tween 20). Staining solution was added to the wells, and the plates were incubated in the dark for 25 min. Bright field images were then obtained using a microscope.

### Colony formation assay

hESCs were differentiated to form EBs for 15 days under non-adherent conditions. EBs were washed with PBS, trypsinized and resuspended in hESC media. hESCs were plated at 10 000 cells per matrigel-coated plate. After 6 days, colonies were fixed and stained for AP. The numbers of colonies were counted and plotted as a percentage of AP-positive colonies per cell plated.

### *In vivo* teratoma formation assay

All procedures involving mice were performed in accordance with institutional guidelines and permissions of the Ethics Committee at the Chinese Academy of Medical Sciences and Peking Union Medical College. Approximately 5 × 10^5^ hNT-2 cells were injected subcutaneously into each 6-week-old male athymic nude (nu/nu) mouse. Xenograft sizes were measured using a vernier caliper at various time points, and the volume of the xenograft was determined using the formula *V*=*(L* × W^2^)/2. Xenograft weights were monitored at the end point.

### Immunofluorescence staining

Cells were washed twice with PBS and fixed with 4% formaldehyde (Sigma Aldrich) in PBS for 30 min at room temperature. Then the cells were permeabilized with 0.1% TritonX-100 in PBS at room temperature for 15 min. Next, they were blocked with 3% BSA (Sigma Aldrich) in PBS for 30 min. Thereafter, the cells were incubated with a primary antibody against CDK2, CDK4, Cyclin D1 (1 : 50; all from Santa Cruz Biotechnology, Inc.), or SSEA4 (1 : 200; Millipore) at 4 °C overnight, nuclei were counterstained with Hoechst 33342 and visualized with an Olympus I × 81 fluorescence microscope.

### Immunohistochemical analysis

Animal or patient specimen tissues were dissected, fixed in 4% PFA (Sigma Aldrich) in PBS overnight, processed, and sectioned into 5 *μ*m slices according to the standard procedures. Then, the sections were permeabilized with 0.1% TritonX-100 in PBS at room temperature for 15 min, and blocked with 3% BSA (Sigma Aldrich) in PBS for 30 min. Then the sections were incubated with a primary antibody against OCT4 or PCNA (1:200; Cell Signaling Technology) at 4 °C overnight. Next, the sections were treated according to the manufacturer's instructions for the ABC detection kit (Vector Laboratories). Negative controls were performed with the omission of the primary antibody. Sections were visualized with an Olympus B × 51 light microscope equipped with an Olympus DP70 camera.

### Small-animal PET

18F-FDG was synthesized automatically with a conventional module used in our clinical work in Peking Union Medical College Hospital. 18 F-FDG was pyrogen-free and qualified for clinical use with a radiochemical purity of more than 99%. All mice were fasted 15 h before microPET scanning. The animals were intraperitoneally injected with 7.4 MBq (0.2 mCi) of 18F-FDG, allowed to regain consciousness and kept at 37 °C until imaging. microPET scans were obtained under standardized conditions using a Siemens Inveon microPET scanner at the end study point. Tissue uptake value was expressed as radioactive count per milliliter (nCi/cc). We measured interscapular BAT maximum uptake value from sagittal images and xenograft mean uptake value from coronal images. The BAT uptake ratio was then calculated for further comparison and analysis.

### Statistical analysis

Two-tailed pairwise Student's *t*-tests were used to analyze the results obtained from two samples with one time point. An analysis of variance (single factor or two factors with replication) was used to compare multiple samples (at one time or several time points). Differences with *P*<0.05 were considered significant. The *P*-values were corrected for multiple testing procedures and to control for type I error rates, calculations were performed with SPSS software version 19.0.

## Abbreviations

hPSCs: human pluripotent stem cells
hESCs: human embryonic stem cells
ECCs: embryonal carcinoma cells
hNT-2: human Tera-2
hMSCs: human adult mesenchymal stem cells
3′UTR: the 3′ untranslated region
NOD/SCID: immunodeficient nonobese diabetic/severe combined immunodeficiency
EB: embryoid body
RA: retinoic acid
