# Supplementary material for: miR-302 regulates pluripotency, teratoma formation and differentiation in stem cells via an AKT1/OCT4-dependent manner
Source: Cell Death Dis. 2016 Jan 28;7(1):e2078–. doi: 10.1038/cddis.2015.383 (PMC4816169; doi:10.1038/cddis.2015.383)
Supplement: Supplementary Information [file cddis2015383x1.doc]

**miR-302 regulates pluripotency, teratoma formation and differentiation in stem cells through an AKT1\OCT4-dependent manner**

**H-L Li^a^,*, J-F Wei^a,b^,*, S-H Wang^a^, L-Y Fan^a^, L Zhu^c^, T-P Li^a^, G Lin^d^, Y Sun^e^, Z-J Sun^c^, J Ding^c^, J Li^a^, Q Han^a, #^, R-C-H Zhao^a,f, #^**

**
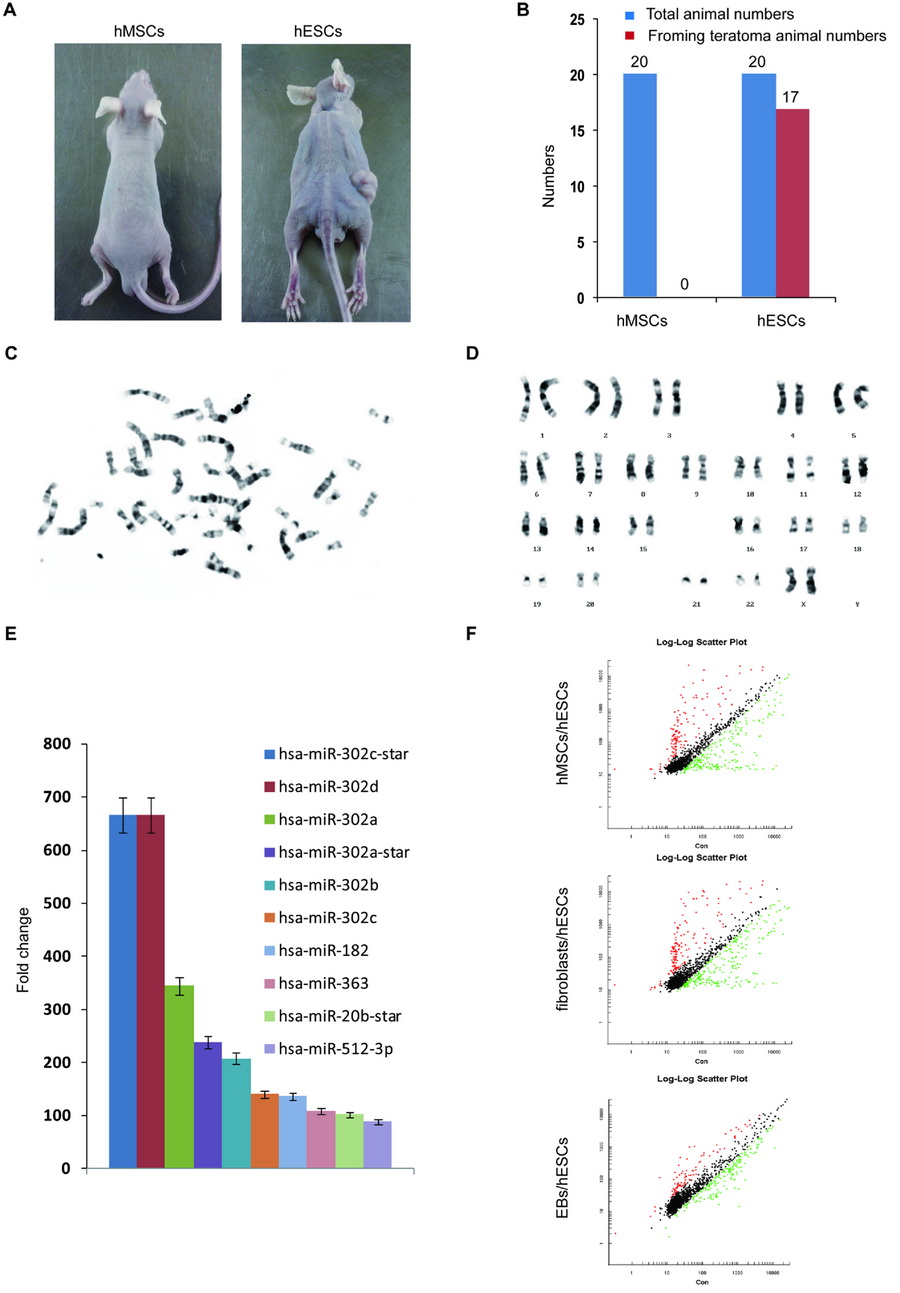
**

**Supplementary Figure S1** Analysis of the karyotype, tumorigenicity and expression profile of miR-302 in hESCs and hMSCs. (**A**, **B**) The in vivo teratoma formation assay was used to compare the tumorigenicity of hMSCs and hESCs. 5×10^5^ cells were injected subcutaneously into immunodeficient mice. Xenografts were detected after transplantation for 60 days. Negative control oligonucleotide antagomir-transfected cells were injected as control (*n* = 20). (**C**, **D**) Karyotype analysis was performed to visualize chromosomes of the hMSCs. (**E**) Fold-change of the top 20 miRNA between hESCs and hMSCs. (**F**) Scatter plot analysis was used to show the similarity between hESCs, hMSCs, fibroblasts and embryoid bodies (EBs). The X-axis and Y-axis fluorescence signal intensity of each of the two samples value coordinates, each data point on the chip represent a gene spot hybridization signal, marked in red and green marker data points denote the B/A ratio value of ≥ 2 and ≤ 0.5, belong to the differentially expressed genes; a black mark indicates ratio value of B/A is between 0.5 and 2.0. There was substantially no difference in expression.


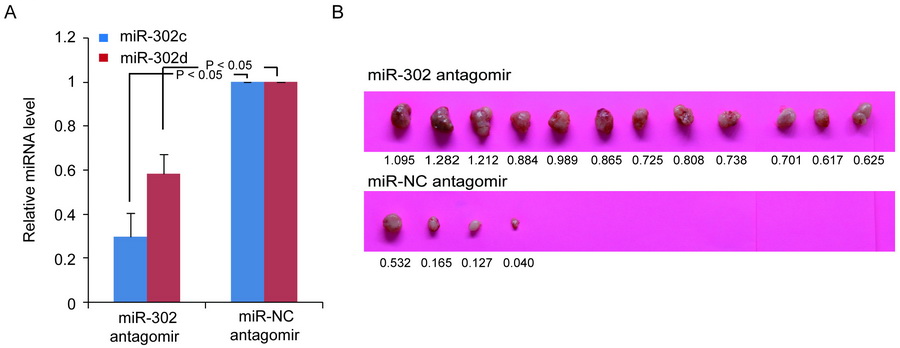


**Supplementary Figure S2** Downregulation of miR-302 suppresses the tumorigenicity of hNT-2 cells *in vivo.* (**A**) TaqMan miRNA qRT-PCR confirmed the inhibitory efficiency of miR-302s antagomir on miR-302 expression. Negative control oligonucleotide was transfected as control, and U6 was used as an internal normalization control. The data are presented as the mean ± S.D. (*n* = 3) and are representative of three independent experiments. (**B***)* The tumor weights were measured at 41 days after inoculation of miR-302s-suppressed hNT-2 and negative control cells (*n = 12*).


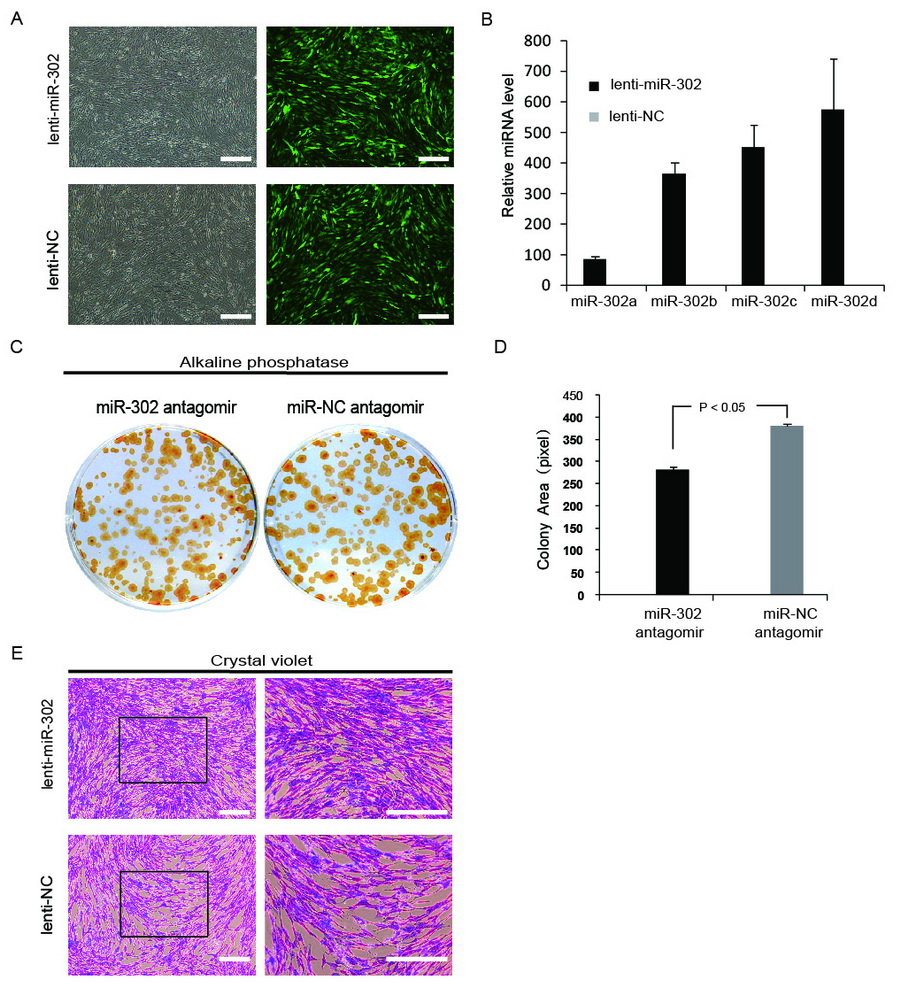


**Supplementary Figure S3** miR-302 regulates the proliferation of hMSCs and hESCs. (**A**) miR-302 was upregulated by expressing the miR-302 precursor from a lentiviral vector (miR-302a, miR-302b, miR-302c and miR-302d in combination). Three days after Lenti-miR-302 viral infection, the GFP-positive hMSC population was isolated by fluorescence-activated cell sorting. The figures are representative of cell condition after cell sorting (left: light, right: fluorescence). Scale bars: 100 *µ*m. (**B**) Comparative quantification of miR-302 expression was performed by stem-loop miRNA qRT-PCR in miR-302s-upregulated hMSCs (lenti-miR-302s) and negative control cells (lenti-NC). U6 was used as an internal normalization control. The data are presented as the mean ± S.D. (*n* = 3) and are representative of three independent experiments. (**C**) AP staining was performed to compare the ability of colony formation of miR-302s-downregulated hESCs to negative control-transfected cells. The panel is representative of three independent assays. (**D**) The mean areas of the AP-positive colonies were calculated and plotted in miR-302s-downregulated hESCs to negative control transfected hESCs. The data are presented as the mean ± S.D. (*n* = 4) and are representative of three independent experiments. (**E**) Crystal violet staining assessed the cell growth when exogenous expression of miR-302 was upregulated by a miR-302s-overexpressing lentiviral vector. A lentiviral vector expressing scrambled sequence was used as a control. The figures are representative of three independent assays. Scale bars: 100 *µ*m.


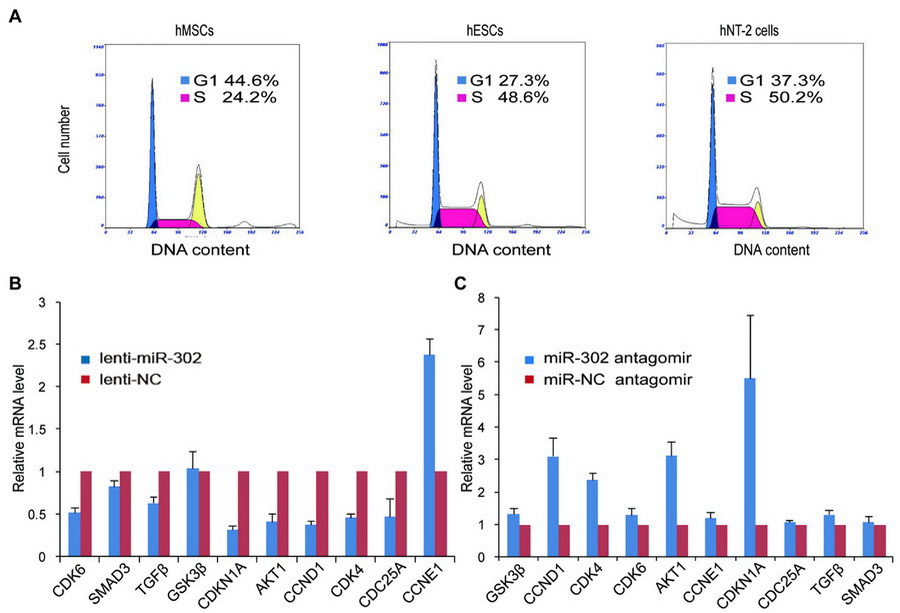


**Supplementary Figure S4** miR-302 affects the cell cycle of human pluripotent and adult stem cells. (**A**) Flow cytometry analysis of the cell cycle of hESCs, hNT-2 and hMSCs. The figures are representative of three independent assays. (**B**) qRT-PCR analysis of the expression levels of cell cycle-related genes in miR-302s-upregulated hMSCs (lenti-miR-302s) and negative control cells (lenti-NC). GAPDH was used as an internal normalization control. The data are presented as the mean ± S.D. (*n* = 3) and are representative of three independent experiments. (**C**) qRT-PCR detected the mRNA levels of cell cycle-related genes in miR-302s antagomir-transfected hNT-2 cells. A negative control oligonucleotide was transfected as control. GAPDH was used as an internal normalization control. The data are presented as the mean ± S.D. (*n* = 3) and are representative of three independent experiments.


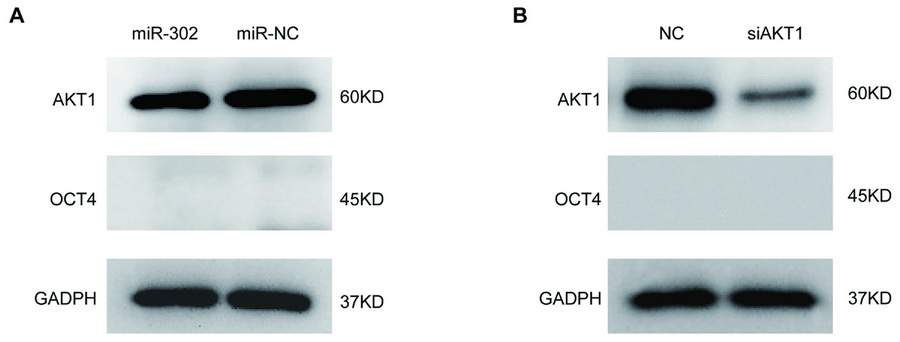


**Supplementary Figure S5** Overexpression of miR-302 affects the expression level of AKT1 and OCT4 in hMSCs. (**A**) Western blot analysis was used to evaluate the expression of AKT1 and OCT4 when exogenous expression of miR-302 was upregulated by a miR-302s-overexpressed lentiviral vector (miR-302s). A lentiviral vector expressing scrambled sequence was used as a control (miR-NC). GAPDH was used as a loading control. The data are representative of three independent experiments. (**B**) Western blot analysis was used to evaluate the expression of AKT1 and OCT4 when exogenous expression of AKT1 was downregulated by special RNA interference. A scrambled sequence was used as a control. GAPDH was used as a loading control. The data are representative of three independent experiments.


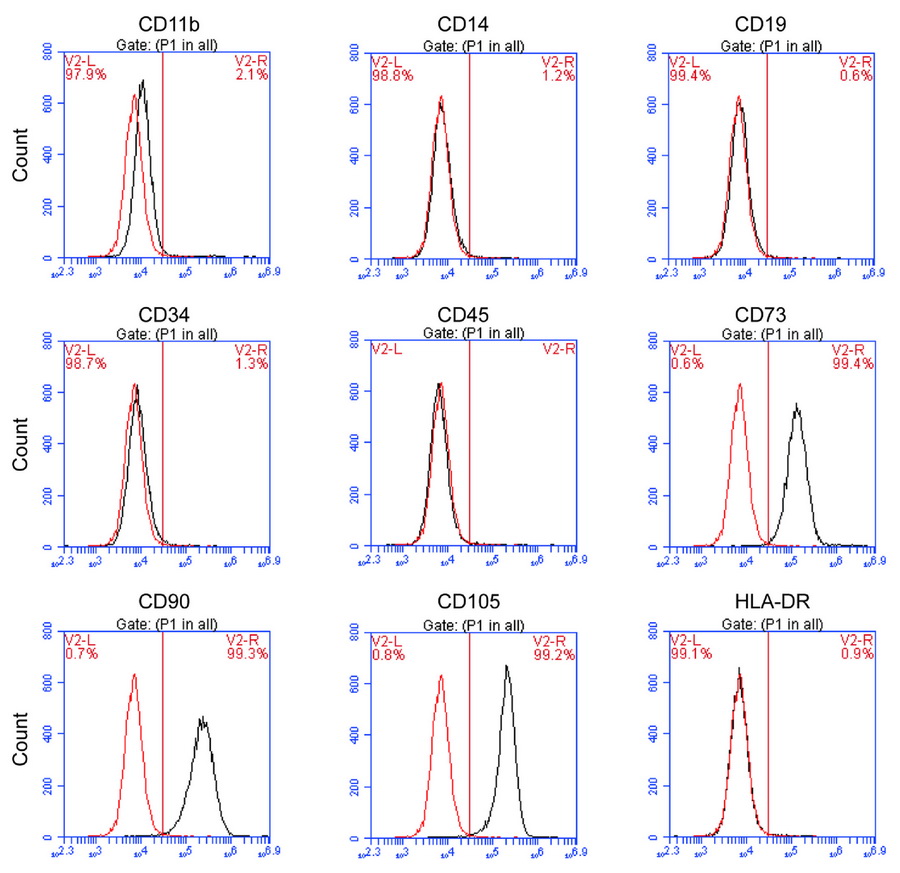


**Supplementary Figure S6** Phenotypic analysis of hMSCs by flow cytometry. Fluorescent intensity histogram of control and experimental antibody stains. Unstained control cells (red line), experimental antibody stained cells (black line).

**Supplementary Table S1**. Fold-change of the top 20 miRNA between hESCs and hMSCs.

| hESCs | | | hMSCs | | | Fibroblasts | | EB |
| --- | --- | --- | --- | --- | --- | --- | --- | --- |
| 1 | 2 | 3 | 4 | 5 | 6 | 7 | 8 | 9 |
| 11831 | 11415 | 10659 | 17.734 | 21.939 | 13.491 | 12.034 | 19.036 | 932.44 |
| 7869.4 | 8388.3 | 11071 | 11.888 | 11.026 | 19.668 | 18.326 | 13.514 | 3578.6 |
| 4804.1 | 4115.7 | 5773.2 | 17.351 | 14.276 | 11.144 | 11.089 | 18.802 | 1401.5 |
| 2788.6 | 3168.3 | 3849.7 | 13.03 | 12.758 | 15.579 | 14.436 | 14.935 | 670.95 |
| 3163.9 | 2216.7 | 3322.2 | 15.159 | 14.047 | 11.777 | 13.303 | 10.655 | 622.46 |
| 1929.3 | 1978.4 | 2285.6 | 15.963 | 12.957 | 14.98 | 15.587 | 18.851 | 477.91 |
| 2502.7 | 2657.2 | 2575.1 | 25.131 | 16.184 | 16.085 | 25.668 | 38.998 | 1430.1 |
| 2087 | 2463.4 | 1134.5 | 19.141 | 12.398 | 18.943 | 16.026 | 16.837 | 4195.2 |
| 2240.5 | 2519.5 | 920.5 | 18.613 | 14.965 | 17.811 | 19.66 | 15.565 | 808.69 |
| 1419.8 | 1344.8 | 2086.9 | 17.611 | 18.789 | 17.15 | 18.086 | 21.694 | 90.485 |

***Supplementary Table S2. mRNA qRT-PCR Primers.***

| Primer | sequence (5'to3') |
| --- | --- |
| AKT1 Forward | AGCGACGTGGCTATTGTGAAG |
| AKT1 Reverse | GTACTCCCCTCGTTTGTGCAG |
| CDK4 Forward | ATGGCTACCTCTCGATATGAGC |
| CDK4 Reverse | TAGGCACCGACACCAATTTCA |
| CDK6 Forward | GCTGACCAGCAGTACGAATG |
| CDK6 Reverse | GCACACATCAAACAACCTGACC |
| CCND1 Forward | GCTGCGAAGTGGAAACCATC |
| CCND1 Reverse | CCTCCTTCTGCACACATTTGAA |
| CDC25A Forward | GGCAGTGATTATGAGCAACCA |
| CDC25A Reverse | CAACAGCTTCTGAGGTAGGGA |
| CCNE1 Forward | GCCAGCCTTGGGACAATAATG |
| CCNE1 Reverse | AGTTTGGGTAAACCCGGTCAT |
| SMAD3 Forward | TGGACGCAGGTTCTCCAAAC |
| SMAD3 Reverse | GTGCTGGGGACATCGGATTC |
| GSK3B Forward | GGCAGCATGAAAGTTAGCAGA |
| GSK3B Reverse | GGCGACCAGTTCTCCTGAATC |
| CDKN1A Forward | TGTCCGTCAGAACCCATGC |
| CDKN1A Reverse | AAAGTCGAAGTTCCATCGCTC |
| TGFB Forward | CAATTCCTGGCGATACCTCAG |
| TGFB Reverse | AGATAACCACTCTGGCGAGTC |
| FBXW7 Forward | CGACGCCGAATTACATCTGTC |
| FBXW7 Reverse | CGTTGAAACTGGGGTTCTATCA |
| FOXA2 Forward | GCGACCCCAAGACCTACAG |
| FOXA2 Reverse | GGTTCTGCCGGTAGAAGGG |
| ZIC1 Forward | CTGGCTGTGGCAAGGTCTTC |
| ZIC1 Reverse | CAGCCCTCAAACTCGCACTT |
| BMP4 Forward | ATGATTCCTGGTAACCGAATGC |
| BMP4 Reverse | CCCCGTCTCAGGTATCAAACT |
| SOX1 Forward | CCTCCGTCCATCCTCTG |
| SOX1 Reverse | AAAGCATCAAACAACCTCAAG |
| FGF8 Forward | CGACCCCTTCGCAAAGCT |
| FGF8 Reverse | GGACTCGAACTCTGCTTCCAAA |
| SOX7 Forward | ACGCCGAGCTCAGCAAGAT |
| SOX7 Reverse | TCCACGTACGGCCTCTTCTG |
| WNT3A Forward | AGGGCACCTCCACCATTTG |
| WNT3A Reverse | GACACTAACACGCCGAAGTCA |
| MEOX1 Forward | GTCCCCCAACTGGCACTTC |
| MEOX1 Reverse | GGTCCCCATTTCCTTGGAACC |
| BRACHYURY Forward | TATGAGCCTCGAATCCACATAGT |
| BRACHYURY Reverse | CCTCGTTCTGATAAGCAGTCAC |
| GATA4 Forward | CGACACCCCAATCTCGATATG |
| GATA4 Reverse | GTTGCACAGATAGTGACCCGT |
| GATA2 Forward | ACTGACGGAGAGCATGAAGAT |
| GATA2 Reverse | CCGGCACATAGGAGGGGTA |
| GAPDH Forward | GGTCACCAGGGCTGCTTTTA |
| GADPH Reverse | GGATCTCGCTCCTGGAAGATG |

***Supplementary Table S3. miRNA qRT-PCR Primers.***

| Primer | sequence (5'to3') |
| --- | --- |
| hsa-miR-302a RT | GTCGTATCCAGTGCAGGGTCCGAGGTATTCGCACTGGATACGACTCACCAA |
| hsa-miR-302b RT | GTCGTATCCAGTGCAGGGTCCGAGGTATTCGCACTGGATACGACCTACTAA |
| hsa-miR-302c RT | GTCGTATCCAGTGCAGGGTCCGAGGTATTCGCACTGGATACGACCCACTGA |
| hsa-miR-302d RT | GTCGTATCCAGTGCAGGGTCCGAGGTATTCGCACTGGATACGACACACTCA |
| hsa-miR-302 Sense | CTGGAGTAAGTGCTTCCATGTTT |
| hsa-miR-302 Antisense | GTGCAGGGTCCGAGGT |
| U6 RT | AAAATATGGAACGCTTCACGAATTTG |
| U6 Sense | CTCGCTTCGGCAGCACATATACT |

***Supplementary Table S4. Dual luciferase reporter gene vectors.***

| Primer | sequence (5'to3') |
| --- | --- |
| AKT1-wt Forward Primer | 5'**CTAGA**AGCCTCCCCCTCAGATGATCTCTCCACGGTAGCACTTGACCTTTTCGACGCTTAACCTTT**GC**3' |
| AKT1-wt Reverse Primer | 5'**GGCCGC**AAAGGTTAAGCGTCGAAAAGGTCAAGTGCTACCGTGGAGAGATCATCTGAGGGGGAGGCT**T**3' |
| AKT1-mut Forward Primer | 5'**CTAGA**AGCCTCCCCCTCAGATGATCTCTCCACGGTCCGGAAAGACCTTTTCGACGCTTAACCTTT**GC**3' |
| AKT1-mut Reverse Primer | 5'**GGCCGC**AAAGGTTAAGCGTCGAAAAGGTCTTTCCGGACCGTGGAGAGATCATCTGAGGGGGAGGCT**T**3' |

***Supplementary Table S5. miRNA sequences.***

| Name | sense（5'-3'） |
| --- | --- |
| hsa-miR-302a | UAAGUGCUUCCAUGUUUUGGUGA |
| hsa-miR-302b | UAAGUGCUUCCAUGUUUUAGUAG |
| hsa-miR-302c | UAAGUGCUUCCAUGUUUCAGUGG |
| hsa-miR-302d | UAAGUGCUUCCAUGUUUGAGUGU |
| hsa-miR-NC | UUCUCCGAACGUGUCACGUTT |
| hsa-miR-302a antagomir | UCACCAAAACAUGGAAGCACUUA |
| hsa-miR-302b antagomir | CUACUAAAACAUGGAAGCACUUA |
| hsa-miR-302c antagomir | CCACUGAAACAUGGAAGCACUUA |
| hsa-miR-302d antagomir | ACACUCAAACAUGGAAGCACUUA |
| hsa-miR-NC antagomir | UUGUACUACACAAAAGUACUG |

***Supplementary Table S6. siRNA sequences.***

| Name | sense（5'-3'） |
| --- | --- |
| AKT1 | GACGGGCACAUUAAGAUCATT |
